# Comparison of Growth Performance, Carcass Properties, Fatty Acid Profile, and Genes Involved in Fat Metabolism in Nanyang and Landrace Pigs

**DOI:** 10.3390/genes15020186

**Published:** 2024-01-30

**Authors:** Jinzhou Zhang, Shuaitao Meng, Heming Wang, Chuankuan Zhang, Zhe Sun, Luyao Huang, Zhiguo Miao

**Affiliations:** College of Animal Science and Veterinary Medicine, Henan Institute of Science and Technology, No. 90, East Section of Hualan Avenue, Xinxiang 453003, China; zhangjz69@126.com (J.Z.); mengshuaitao2001@163.com (S.M.); sqheming@163.com (H.W.); chuankuan0526@163.com (C.Z.); sz18437911393@163.com (Z.S.); huangluyao_12@163.com (L.H.)

**Keywords:** pigs, lipogenic enzyme, fatty acid profile, fat deposition, subcutaneous fat tissue, gene expression

## Abstract

This study compared the growth, carcass properties, fatty acid profile, lipid-producing enzyme activity, and expression pattern of genes involved in fat metabolism in Nanyang and Landrace pigs. In the study, 32 Nanyang (22.16 ± 0.59 kg) and 32 Landrace barrows (21.37 ± 0.57 kg) were selected and divided into two groups, each with eight pens and four pigs per pen. The trial period lasted 90 days. The findings showed that the Nanyang pigs had lower average daily weight gain and lean percentage and higher average backfat thickness and lipogenic enzyme activities, including for acetyl-CoA carboxylase, glucose-6-phosphate dehydrogenase, malic enzyme, and fatty acid synthase, than the Landrace pigs. A total of 14 long-chain fatty acids were detected using HPLC-MS, in which it was found that the levels of C14:0, C18:1n-9, C20:1n-9, C20:4n-6, and MUFA were up-regulated and C18:2n-6, C18:3n-3, PUFA n6, n3/n6, and total PUFA were down-regulated in the Nanyang pigs. Moreover, the mRNA levels for genes involved in fat metabolism, *ME1*, *FAS*, and *LPL*, were higher and the expression of *SREBP1* mRNA was lower in the Nanyang pigs. Our results suggest genetic differences between the pig breeds in terms of growth, carcass traits, lipogenic enzyme activities, fatty acid profile, and the mRNA expression of genes involved in fat metabolism in subcutaneous fat tissue, which may provide a basis for high-quality pork production. Further studies are needed to investigate the regulation of lipid metabolism.

## 1. Introduction

In China, with the increase in living standards, high-quality pork is highly favored by the consumer market. However, the current market supply of high-quality pork cannot meet the needs of consumers. Fat deposition is a vital factor affecting pork quality and is related to meat appearance, flavor, juiciness, tenderness, and processing characteristics, as well as human health [1]. It is well known that cardiovascular disease is closely related to dietary fat intake and fatty acid composition [2]. Animal body fat, including subcutaneous, abdominal, intermuscular, and intramuscular fat, is distributed in different parts of the body. Therefore, exploring the molecular mechanism of fat deposition has great significance for high-quality pork production. In addition to nutritional levels, age, weight, gender, and environment, fat deposition is influenced by the breeds of the pigs [3,4,5]. Local pigs are known to produce high-quality pork due to having a higher capacity for fat deposition than modern commercial breeds [6]. Zhao et al. [7] found that the terminal body weight (BW) and the hormone-sensitive lipase (HSL) activity in longissimus dorsi muscles were lower, and the backfat thickness, lean fat weight, and fatty acid synthase (FAS) activity in longissimus dorsi muscles were greater in Mashen pigs than in Large White pigs. The mRNA expression of CCAAT/enhancer-binding protein α (*C/EBPα*), *C/EBPβ*, and peroxisome proliferator-activated receptor γ (*PPARγ*) in longissimus dorsi muscles was increased in Mashen pigs compared to Large White pigs. There was no difference in the sterol regulatory element-binding protein 1 (*SREBP1*) mRNA levels in longissimus lumborum muscles between these two breeds. Another study evaluated the effects of genetics on the fat amount and fatty acid composition in Iberian and Landrace × Large White pigs and found that the swine genetic type had an important influence on the fatty acid profile of the outer and inner backfat layers. The monounsaturated fatty acid (MUFA) level in Iberian pigs at 115 kg in the two layers was greater than that in Landrace × Large White pigs [8]. Zhang et al. [9] found that Bamei pigs had a greater capacity for fat deposition than Landrace pigs, owing to the function of pre-adipocytes; the adipogenesis induced by glucose was greater in Bamei pigs than in Landrace pigs. Although there have been several studies on fat deposition in different varieties of pigs, a deeper understanding of the underlying hereditary effects of fat deposition in pigs can be achieved through further in-depth study.

The Nanyang pig is an ancient breed distributed in the Nanyang area of Henan Province in China. It is early maturing, fattens easily, and is a medium-sized meat fat dual-use-type breed. It is a Chinese local pig breed famous for its stable genetic characteristics, strong and robust physique, strong adaptability, disease resistance, and delicious meat. A previous study carried out proteome analyses on longissimus dorsi and backfat tissues from two groups of Nanyang pigs with different fat deposition efficiencies and identified 15 candidate genes determining lipid deposition, in which *FASN*, *CAT*, and *SLC25A20* were the most prominent; the findings indicated that the Nanyang pig is an optimal animal model to investigate high meat quality and fast fat deposition mechanisms in pigs [10]. The Landrace pig is a common commercial variety known for its high growth speed and lean carcass. In this study, we posit that a better understanding of these two breeds’ differences would be helpful to clarify the distinction in fat deposition mechanisms in pigs of different genetic backgrounds and provide a basis for high-quality pork production. Therefore, we investigate the differences in the growth performance, carcass properties, fatty acid profile, lipid-producing enzyme activity, and expression of genes involved in fat metabolism between Nanyang and Landrace pigs.

## 2. Materials and Methods

### 2.1. Animals, Experimental Design, Diets, and Management

The pig-rearing procedure was approved by the Animal Protection and Utilization Committee of Henan Institute of Science and Technology (No. 2020HIST004, 02/04/2020, Xinxiang, China). A total of 64 barrows (32 Nanyang pigs, average body weight: 22.16 ± 0.59 kg, and 32 Landrace pigs, average body weight: 21.37 ± 0.57 kg) were selected from Xinda Husbandry Development Co., Ltd. (Luoyang, Henan Province, China). Each variety had 8 pens, with 4 pigs per pen. The pigs in the 16 pens were reared in a hog house and fed with the same commodity feed; the ingredients and nutrient contents are presented in Table 1. During the experiment, the pigs were free to feed and drink water ad libitum. Daily management was carried out in accordance with the company’s procedures. After 5 days of pre-testing, the trial period lasted 90 days. In the whole experiment, the feed consumed and the residual feed were measured daily, and then the initial BW, final BW, average daily weight gain (ADG), average daily feed intake (ADFI), and feed/gain ratio (F/G) of the pigs were determined.

### 2.2. Sample Collection

At the end of the experiment, 16 pigs (2 pigs per pen, close to the average weight of their breed group) in each breed were selected; after weighing, the animals were fasted for twelve hours, given free access to water, and then slaughtered after electrical stunning at a commercial abattoir according to Chinese standard industry procedures. After slaughter, each carcass was weighed, and then the average backfat depth at the 1st rib, 1st lumbar, and last lumbar vertebrae was calculated using a sliding caliper in the midline at the right carcass sides [11]. The loin muscle area was measured at the transversal surface of the longissimus lumborum between the last thoracic vertebra and first lumbar vertebra using a sliding caliper (width × thickness × 0.7, cm^2^). The lean meat percent was calculated according to the formula (lean weight ÷ carcass weight × 100%). Approximately 100 g of subcutaneous fat from the outer backfat layer at the 13th rib on the right side of the carcass was obtained immediately post mortem and kept at −80 °C for further testing of the fatty acid profile, lipid-producing enzyme activity, and expression of genes involved in fat metabolism [12].

### 2.3. Assay of Lipid-Producing Enzyme Activities in Subcutaneous Fat Tissue

The activities of acetyl-CoA carboxylase (ACC, EC6.4.1.2), malic enzyme (ME, EC 1.1.1.40), glucose-6-phosphate dehydrogenase (G6PDH, EC 1.1.1.49), and FAS (EC 2.3.1.85) in the subcutaneous fat tissue were determined according to Gerfault et al. [13]. All of the enzyme assays were determined in triplicate. Then, 1 g of frozen subsample from the collected subcutaneous fat tissue was immediately homogenized in 0.25 M sucrose solution on ice, and then centrifuged for 30 min at 30,000× *g* and 4 °C. Next, the supernatants were collected to detect the lipogenic enzyme activities using the colorimetric method according to the producer’s manual (Nanjing Jiancheng Bioengineering Institute, Nanjing, China). One unit of G6PDH and ME activity was determined as the enzymes production of 1 nmol nicotinamide adenine dinucleotide phosphate (NADPH) min^−1^·g sample^−1^, resulting from the reduction in NADP^+^. ACC reacts with acetyl-CoA and ATP: the products are malonyl-CoA, ADP, and inorganic phosphorus, and its activity was defined as the enzyme generating 1 nmol of PO_4_^3−^·g sample^−1^ per hour as one unit. The FAS activity was defined as 1 nmol of NADPH oxidized to NADP^+^ min^−1^·g sample^−1^ as one unit.

### 2.4. Assay of Fatty Acid Profile in Subcutaneous Fat Tissue

Fatty acid samples from the subcutaneous fat tissue (0.05 g) were prepared according to Folch et al. [14], put into 10 mL centrifuge tubes, and obtained by mixing a solution of 5 mL chloroform/methanol (2:1 *v*/*v*) for two hours after homogenizing. Next, 5 mL of distilled water was added and mixed well, and the mixed solution was centrifuged for 5 min at 3000× *g* and 25 °C. Finally, the supernatants were discarded, and the lower solutions were dried with negative pressure. The methylation of fatty acids followed this procedure: 1 mL of n-hexane was added and swung for 25 min at 40 °C, and then 1 mL KOH-methanol solution (0.4 mol/L) was added; after standing for 25 min, the mixtures were shaken for 2 h. Then, 2 mL of deionized water was added and blended, and centrifugation for 5 min at 3000× *g* was carried out. The supernatant was collected and detected using high-performance liquid chromatography–mass spectrometry (HPLC-MS) for the analysis of the fatty acid profile. The HPLC-MS was performed on a SCIEX QTRAP 4500 mass spectrometer (Applied Biosystems, Framingham, MA, USA) coupled with an Agilent 1260 Infinity HPLC system (Agilent Technologies, Santa Clara, CA, USA). The mobile phases consisted of acetonitrile (A) and isopropanol (B). The procedure of gradient elution (0–10 min, 100% B; 11–30 min, 70% B; 31–120 min, 50% B) was performed at a flow rate of 0.2 mL/min; 10 μL of the sample was injected into the chromatographic system. Mass spectrometry was carried out under atmospheric pressure chemical ionization in positive ion mode (APCI^+^); spray voltage, 1.5 kV; capillary temperature, 300 °C; MS scanning range, 50−1200 amu; scanning rate, 50 amu/s. The result of the fatty acid analysis was described by g fatty acids/100 g of detected fatty acid methyl esters. All of the fatty acid assays were conducted in triplicate.

### 2.5. Assay of Quantitative Real-Time PCR

The isolation of total RNA from the subcutaneous fat tissues was performed with TRIzol (Invitrogen, Waltham, MA, USA) according to the producer’s manual. The quantity and quality of the obtained RNA were evaluated with a NanoPhotometer^®^ spectrophotometer (Implen, Westlake Village, CA, USA; 1.8 < OD260/OD280 < 2.0). The cDNA synthesis was performed with AMV reverse transcriptase (Promega, Madison, WI, USA). The mRNAs of adipose triglyceride lipase (*ATGL*), *SREBP1*, leptin (*LEP*), malic enzyme 1 (*ME1*), *FAS*, hormone-sensitive lipase (*HSL*), and lipoprotein lipase (*LPL*) were selected for the detection of the expression profiles of genes involved in fat metabolism. The primer pairs were designed for quantitative real-time PCR (qRT-PCR), as presented in Table 2. The qRT-PCRs were performed with the QuantiFast SYBR^®^ Green RT-PCR Kit (Qiagen, Hilden, Germany) according to the producer’s manual. The mRNA expression level of these genes was determined using the 2^–△△Ct^ method [15], with β-actin as a housekeeping gene. The assays of the genes involved in fat metabolism were carried out in triplicate.

### 2.6. Statistical Analysis

All data were analyzed using an independent-group two-tailed *t*-test in SPSS 26.0 (IBM Corp., Armonk, NY, USA), and significant differences between breeds were indicated by *p* < 0.05.

## 3. Results

### 3.1. Growth Performance and Carcass Traits 

The growth performance data of the Nanyang and Landrace pigs are presented in Table 3. There was no significant difference in the initial BW (0.79 kg; *p* = 0.172) or ADFI (114.9 g/d; *p* = 0.116) between the Nanyang and Landrace pigs. Compared to Landrace, the Nanyang pigs had a significantly lower final BW (20.04 kg; *p* < 0.001) and ADG (231.5 g/d; *p* < 0.001), and a significantly higher F/G (0.95; *p* = 0.001).

As presented in Table 4, the carcass weight (15.77 kg; *p* = 0.003), loin muscle area (18.07 cm^2^; *p* = 0.001), and lean percentage (10.99%; *p* = 0.005) in the Nanyang pigs were lower than those in the Landrace pigs. The average backfat thickness in the Nanyang pigs was higher than that in the Landrace pigs (6.76 mm; *p* = 0.003).

### 3.2. Assay of Lipid-Producing Enzyme Activities in Subcutaneous Fat Tissue

As presented in Table 5, the lipid-producing enzyme activities of ACC (6.39 U; *p* = 0.010), G6PDH (132.28 U; *p* = 0.001), ME (130.88 U; *p* = 0.002), and FAS (17.24 U; *p* = 0.001) were up-regulated in the Nanyang pigs compared to the Landrace pigs.

### 3.3. Assay of Fatty Acid Profile of Subcutaneous Fat Tissue

A total of 14 long-chain fatty acids were detected using HPLC-MS (Table 6). The levels of C14:0 (0.20 g; *p* = 0.047), C18:1n-9 (2.34 g; *p* = 0.011), C20:1n-9 (0.30 g; *p* = 0.040), C20:4n-6 (0.35 g; *p* = 0.023), and MUFA (3.14 g; *p* = 0.002) were greater in the Nanyang pigs than in the Landrace pigs, whereas the C18:2n-6 (5.14 g; *p* = 0.003), C18:3n-3 (0.08 g; *p* = 0.011), PUFA n6 (4.82 g; *p* < 0.001), PUFA n3/n6 (2.82 g; *p* < 0.001), and total PUFA (4.89 g; *p* = 0.005) levels were lower in the Nanyang pigs than in the Landrace pigs. The levels of PUFA n3 (0.09 g; *p* = 0.351) and saturated fatty acids (SFA, 1.75 g; *p* = 0.097) did not differ between the breeds.

### 3.4. Assay of Expression of mRNAs Involved in Fat Metabolism

**Figure 1:**
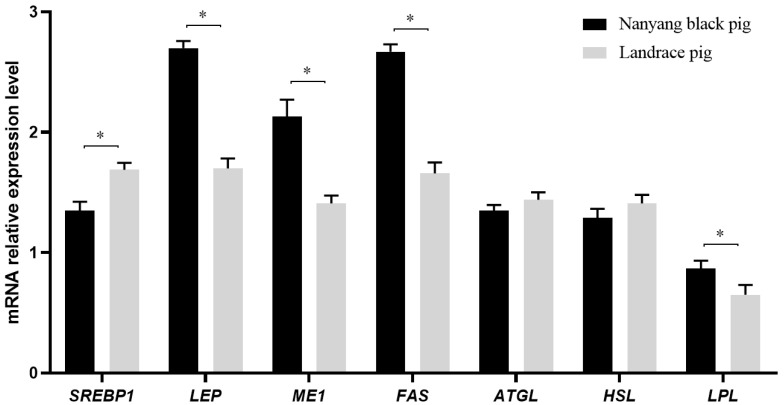
Expression levels of mRNAs involved in fat metabolism in subcutaneous fat tissue in Nanyang and Landrace pigs. The mRNA expressions of *SREBP1*, *LEP*, *ME1*, *FAS*, *ATGL*, *HSL*, and *LPL* in the subcutaneous fat tissue of Nanyang pigs and Landrace pigs were detected using qRT-PCR. The values with an asterisk (*) mean significant differences (*p* < 0.05). *ATGL*, adipose triglyceride lipase; *FAS*, fatty acid synthase; *HSL*, hormone-sensitive lipase; *LEP*, leptin; *LPL*, lipoprotein lipase; *ME1*, malic enzyme 1; *SREBP1*, sterol regulatory element-binding transcription factor 1.

As presented in Figure 1, the expression of *LEP* (1.00; *p* = 0.001), *ME1* (0.72; *p* = 0.001), *FAS* (1.01; *p* = 0.002), and *LPL* (0.18; *p* = 0.005) was up-regulated in the Nanyang pigs compared to the Landrace pigs. *SREBP1* expression was down-regulated in the Nanyang pigs compared to the Landrace pigs (0.34; *p* = 0.003) and the expression of *ATGL* (0.09; *p* = 0.058) and *HSL* (0.12; *p* = 0.065) did not differ between the breeds.

## 4. Discussion

We found that the Nanyang pigs had lower final BW, ADG, carcass weight, loin muscle area, and lean percentage compared with the Landrace pigs, but had greater F/G and average backfat thickness. These findings are consistent with those in previous research [9,16,17,18,19] in which native pig breeds (Iberian, Creole, Bamei, Iberian, Meishan) displayed a lower tendency for growth and better capacity for fat deposition than modern pig breeds (Landrace, Large White, Duroc). Miao et al. [20] studied Jinhua and Landrace pigs and found a lower carcass lean percentage and greater carcass fat frequency in Jinhua pigs than in Landrace pigs. Gispert et al. [21] studied five breeds of pigs (Large White, Landrace, Duroc, Pietrain, and Meishan) and discovered that pigs from the Chinese local breed, Meishan, had lower growth potential and carcass lean meat. Similar results were found for the growth and carcass traits of pigs with breed-specific patterns when Iberian [17], Creole [16], Alentejana [22], and Bamei [9] pigs were compared with modern pig breeds.

Lipogenesis is principally carried out in the fat tissue of pigs and depends on the activities of enzymes [1,23,24]. In this research, we identified that the Nanyang pigs had higher lipogenic enzyme activities, such as ACC, G6PDH, ME, and FAS activity, than those of the Landrace pigs. ACC is an important rate-limiting enzyme for malonyl coenzyme A synthesis, which is the first phase of adipose biosynthesis, and is also an enzyme that effectively inhibits mitochondrial fatty acid oxidation through the regulation of carnitine palmitoyltransferase-1 (CPT-1) [25]. FAS is the core enzyme needed for the transformation of carbohydrates into fatty acids, and it catalyzes the transformation of acetyl coenzyme A and malonyl coenzyme A into fatty acids in the presence of NADPH [26]. ME and G6PDH are important enzymes required to provide NADPH for the reductive biosynthesis of fatty acids [27,28]. Freire et al. found that the ACC (three- and nine-fold), ME (six- and five-fold), and G6PDH (four- and five-fold) increased significantly more in the dorsal subcutaneous and perirenal fat of Alentejano piglets than in Large White piglets [29]. Another study showed that the function of lipid-producing enzymes (FAS, ME, and G6PDH) was greater in the subcutaneous backfat tissue of Iberian pigs than that in Landrace × Large White pigs [30]. The breed-specific differences in the key lipogenic enzymes in this study are similar to those found in previous studies [26,27,28,29,30].

Our study revealed significant differences between the Nanyang and Landrace pigs in terms of MUFAs and PUFAs. There were significantly more MUFAs in the Nanyang pigs than in the Landrace pigs, mainly because of the addition of oleic acid (C18:1 n-9). The Nanyang pigs had significantly lower PUFA levels compared to the Landrace pigs, mainly due to the variations in linoleic acid (C18:2 n-6). These findings are in agreement with those in Barea’s research, which reported that Iberian barrows had higher MUFA levels and lower SFA levels than Landrace × Large White barrows in the subcutaneous outer fat both at 50 and 115 kg of body weight [8]. The MUFA content was greater in Iberian pigs at 115 kg body weight in both the outer and inner backfat, and PUFAs and C18:2 n-6 were higher in both layers (along with body weight) in Landrace × Large White barrows [8]. Another study found a higher content of MUFAs and lower PUFA levels in Pulawska pigs than those in Polish Landrace pigs, demonstrating that the genetic type had a vital function in the fatty acid profile [31]. The PUFA n-6/n-3 ratio is a valuable indicator for evaluating the nutritional quality of meat. A previous study showed that a low PUFA n-6/n-3 ratio (4:1) diet increased piglets’ weaning survival rate and weight gain, and improved the piglets’ health [32]. In this paper, interestingly, we found that the Nanyang pigs had a lower PUFA n-6/n-3 ratio (6.90:1) in subcutaneous backfat tissue than the Landrace pigs (9.72:1), which may mean that the former has higher nutritive value. However, no significant differences in the PUFA n-3 or SFAs between the Nanyang and Landrace pigs were detected in this study.

We found that the contents of *LEP*, *ME1*, *FAS*, and *LPL* mRNA were significantly up-regulated and the *SREBP1* mRNA contents were significantly down-regulated in the Nanyang pigs compared to the Landrace pigs, with no significant difference in the content of *ATGL* and *HSL* mRNA being observed between them. Benítez et al. found that *LEP*, *ME1*, and *FAS* had higher expression and *ATGL* lower expression in the subcutaneous adipose tissue from Iberian pigs compared to Duroc pigs, showing that the Iberian pigs had a more stable expression of lipogenic genes [19]. Xing et al. identified the differential expression of genes in backfat tissue between Songliao and Landrace pigs using high-throughput sequencing, and found that the *ACC* increased significantly and *LEP* decreased significantly in Songliao pigs compared to Landrace pigs [33]. In another study, the *SREBP-1c*, *FAS*, and *ACC1* (on d 10) mRNA expression levels were increased in the subcutaneous fat of Bamei pigs compared to in Landrace pigs [9]. In this paper, genes involved in fat metabolism were identified, and they may explain the phenotypic differences observed between Nanyang and Landrace pigs.

## 5. Conclusions

In the present paper, we demonstrated the phenotypic differences between Nanyang and Landrace pigs in terms of growth, carcass traits, fatty acid profile, lipogenic enzyme activities, and expression of mRNAs involved in fat metabolism in subcutaneous fat tissue, which might explain the differences in fat deposition linked to different breeds of pigs. This paper provides references for the future breeding of native breeds and for high-quality pork production. Further studies to elucidate the molecular mechanisms of lipid metabolism, so as to reduce the fatness of Nanyang pigs, have practical implications.

## Figures and Tables

**Table 1:** Composition and nutritional content of basic diet.

Items	Content	
	20–60 kg	61–100 kg
Ingredient (%)		
Corn	67.4	78.3
Soybean meal	19.5	15
Wheat bran	8.53	3.95
Limestone	0.89	0.55
Soybean oil	2.38	0.9
NaCl	0.3	0.3
Premix ^1^	1.00	1.00
Total	100.00	100.00
Nutrient level ^2^		
Metabolic energy ME (MJ·kg^−1^)	14.21	14.20
Crude protein CP (%)	16.16	14.17
Calcium Ca (%)	0.65	0.6
Total phosphorus TP (%)	0.45	0.35
Available phosphorus AP (%)	0.31	0.25
Met + Cys (%)	0.57	0.42
Lysine Lys (%)	1.17	1.03
SID lysine (%)	1.05	0.93

^1^ The premix supplied per kg of diet: 80 mg Zn, 10 mg Cu, 30 mg Mn, 80 mg Fe, 0.3 mg Se, and 0.5 mg I; 5850 IU vitamin A, 1251 IU vitamin D_3_, 20 IU vitamin E, 3 mg vitamin B_1_, 1.5 mg vitamin B_6_, 20 mg vitamin B_12_, 1.86 mg vitamin K_3_, 18 mg pantothenic acid, 3.6 mg riboflavin, 56 mg of choline, and 26 mg niacin. ^2^ Nutrient level was determined according to the Tables of Feed Composition and Nutritive Values in China (2015, Twenty-Sixth Edition). Abbreviations: Met + Cys, methionine and cysteine; SID lysine, standardized ileal-digestible lysine.

**Table 2:** Design of primer pairs for genes involved in fat metabolism.

Gene	ID	Sequence (5′-3′)	Product Length (bp)
*β-actin*	XM_021086047	ACCTTCTACAACGAGCTGCGTG GTCTCCGGAGTCCATCACGATG	207
*SREBP1*	NM_214157	CTGGTGGTGGGCACGGAGGC TGCCACTGCCACCGCTGCTGC	316
*LEP*	U63540	ACTGCCCCAGAAGCACATCC ATGGAGCCCAGGGATGAAG	232
*ME1*	XM_001924333	ACTGATGGAGAACGTATTC ACTGCCTCCATGAATTCATCC	245
*FAS*	EF589048	TGTCTGGGTGGGTGTGAGCAG GAGTTAGGCTTCAGCAGGAC	270
*ATGL*	EU047807	TTCGCGGGTTGCGGCTTCCTC GATGGTCTTCACCAGGTTGAA	249
*HSL*	AY686758	CACAGCATGGACCTGCGCACA TCGAAGAGGTGTGCCACACTC	191
*LPL*	AY686761	AGATGGAGAGCAAAGCCCTGC GGGACCCAACTTTCATACAT	289

*ATGL*, adipose triglyceride lipase; *FAS*, fatty acid synthase; *HSL*, hormone-sensitive lipase; *LEP*, leptin; *LPL*, lipoprotein lipase; *ME1*, malic enzyme 1; *SREBP1*, sterol regulatory element-binding transcription factor 1.

**Table 3:** Impacts of genetic type on growth performance in Nanyang and Landrace pigs.

Genetic Type				
Item	Nanyang Pigs	Landrace Pigs	SEM	*p*-Value
IBW (kg)	22.16	21.37	0.677	0.172
FBW (kg)	74.85 ^b^	94.89 ^a^	2.621	<0.001
ADG (g/d)	585.4 ^b^	816.9 ^a^	29.587	<0.001
ADFI (g/d)	2254.1	2369.0	90.414	0.116
F/G	3.85 ^a^	2.90 ^b^	0.126	0.001

In the same line, values labeled with different lowercase letters mean significant differences (*p* < 0.05). ADFI, average daily feed intake; ADG, average daily weight gain; IBW, initial body weight; FBW, final body weight; F/G, feed/gain ratio; SEM, standard error of the mean (*n* = 32).

**Table 4:** Effects of genetic type on carcass properties in Nanyang and Landrace pigs.

Genetic Type				
Item	Nanyang Pigs	Landrace Pigs	SEM	*p*-Value
Carcass weight (kg)	52.50 ^b^	68.27 ^a^	8.795	0.003
Loin muscle area (cm^2^)	27.70 ^b^	45.77 ^a^	4.273	0.001
Lean percentage (%)	50.39 ^b^	61.38 ^a^	6.393	0.005
Average backfat thickness ^1^ (mm)	22.11 ^a^	15.35 ^b^	3.883	0.003

^1^ Average backfat thickness was determined on the 1st rib, 1st lumbar, and last lumbar vertebrae of the right side of each carcass in the midline in pigs. In the same line, values labeled with different lowercase letters mean significant differences (*p* < 0.05). SEM, standard error of the mean (*n* = 16).

**Table 5:** Effects of genetic type on the lipid-producing enzyme activities of subcutaneous fat tissue in Nanyang and Landrace pigs.

Genetic Type				
Item	Nanyang Pigs	Landrace Pigs	SEM	*p*-Value
ACC ^1^	23.21 ^a^	16.82 ^b^	1.817	0.010
G6PDH ^2^	345.91 ^a^	213.63 ^b^	12.245	0.001
ME ^3^	452.72 ^a^	321.84 ^b^	18.487	0.002
FAS ^4^	51.27 ^a^	34.03 ^b^	2.313	0.001

^1^ ACC was defined as the enzyme generating 1 nmol of PO4^3−^·g sample-1 per hour as one unit. ^2,3^ G6PDH and ME were defined as the enzyme producing 1 nmol NADPH min^−1^·g sample^−1^ as one unit. ^4^ FAS was defined as 1 nmol of NADPH oxidized per min^−1^·g sample^−1^ as one unit. In the same line, values labeled with different lowercase letters mean significant differences (*p* < 0.05). ACC, acetyl-CoA-carboxylase; FAS, fatty acid synthase; G6PDH, glucose-6-phosphate dehydrogenase; ME, malic enzyme; SEM, standard error of the mean (*n* = 16).

**Table 6:** Effects of genetic type on the fatty acid profile (g fatty acids/100 g of detected fatty acid methyl esters) of subcutaneous fat tissue in Nanyang and Landrace pigs.

Genetic Type				
Item	Nanyang Pigs	Landrace Pigs	SEM	*p*-Value
C14:0	1.52 ^a^	1.32 ^b^	0.136	0.047
C16:0	27.57	25.76	0.804	0.052
C16:1 n-7	3.85	3.27	0.577	0.252
C18:0	13.66	13.94	0.446	0.494
C18:1 n-7	3.24	3.33	0.188	0.603
C18:1 n-9	37.56 ^a^	35.22 ^b^	1.407	0.011
C18:2 n-6	7.28 ^b^	12.42 ^a^	2.957	0.003
C18:3 n-3	1.34 ^b^	1.42 ^a^	0.047	0.014
C20:0	0.27	0.24	0.024	0.284
C20:1 n-9	1.26 ^a^	0.96 ^b^	0.177	0.040
C20:3 n-6	0.06	0.07	0.016	0.670
C20:4 n-6	2.18 ^a^	1.83 ^b^	0.219	0.023
C22:4 n-6	0.14	0.16	0.014	0.294
C22:5 n-3	0.06	0.07	0.012	0.349
Total SFA	43.02	41.27	0.309	0.097
Total MUFA	45.91 ^a^	42.77 ^b^	1.787	0.002
PUFA n3	1.40	1.49	0.113	0.351
PUFA n6	9.66 ^b^	14.48 ^a^	0.516	<0.001
PUFA n3/n6	6.90 ^b^	9.72 ^a^	0.375	<0.001
Total PUFA	11.07 ^b^	15.96 ^a^	1.440	0.005

In the same line, values labeled with different lowercase letters mean significant differences (*p* < 0.05). MUFA, monounsaturated fatty acids; PUFA, polyunsaturated fatty acids; SEM, standard error of the mean (*n* = 16); SFA, saturated fatty acids.

## Data Availability

The data sets that support the findings of this study are available from the corresponding author upon reasonable request.
